# Identifying Twitter users who repost unreliable news sources with linguistic information

**DOI:** 10.7717/peerj-cs.325

**Published:** 2020-12-14

**Authors:** Yida Mu, Nikolaos Aletras

**Affiliations:** Department of Computer Science, The University of Sheffield, Sheffield, United Kingdom

**Keywords:** Social Media, Natural Language Processing, Disinformation

## Abstract

Social media has become a popular source for online news consumption with millions of users worldwide. However, it has become a primary platform for spreading disinformation with severe societal implications. Automatically identifying social media users that are likely to propagate posts from handles of unreliable news sources sometime in the future is of utmost importance for early detection and prevention of disinformation diffusion in a network, and has yet to be explored. To that end, we present a novel task for predicting whether a user will repost content from Twitter handles of unreliable news sources by leveraging linguistic information from the user’s own posts. We develop a new dataset of approximately 6.2K Twitter users mapped into two categories: (1) those that have reposted content from unreliable news sources; and (2) those that repost content only from reliable sources. For our task, we evaluate a battery of supervised machine learning models as well as state-of-the-art neural models, achieving up to 79.7 macro F1. In addition, our linguistic feature analysis uncovers differences in language use and style between the two user categories.

## Introduction

Social media has become an important source for online news consumption, widely adopted by news outlets, individual journalists and end users (Hermida et al., 2012; Kalsnes & Larsson, 2018). The use of social media enhances civic engagement and political participation offering a direct way of communication with millions of users worldwide (Bennett, 2008; Gil de Zúñiga, Jung & Valenzuela, 2012).

A widespread phenomenon in social media platforms is the generation and dissemination of unreliable content (e.g., fabricated or deceptive information, exaggerated headlines, pseudoscience, propaganda) by particular news outlets that typically act as disinformation diffusion sources. Diffusion of disinformation in social media typically begins when a news source publishes a story that subsequently is propagated by users via reposting (e.g., retweeting, sharing) it to their personal networks of friends. It has been observed that disinformation propagates faster compared to credible information amongst users in social media (Vosoughi, Roy & Aral, 2018; Lazer et al., 2018). Furthermore, when a user comes across an unreliable story once, it is enough to increase their later perception of its accuracy (Pennycook, Cannon & Rand, 2018). Media that disseminate unreliable content often aim to manipulate people’s opinion and influence election results which has implications to political stability worldwide (Allcott & Gentzkow, 2017; Humprecht, 2018).

Previous studies suggest that factors positively associated with the sharing unreliable news posts on social network include psychological factors (e.g., online trust, self-disclosure, fear of missing out, and ideological extremity) and political orientation (e.g., right-leaning) (Shu et al., 2019; Talwar et al., 2019; Hopp, Ferrucci & Vargo, 2020). In this study, we investigate whether user language information can help identify who will repost items from Twitter handles of unreliable news sources. To test this hypothesis, we define a new classification task seeking to predict whether a user is likely to repost content from *unreliable news sources* given all the history of the user’s posts up to the first repost of a news item, i.e., *before they actually do it*. *Early detection* of users that are likely to repost content from unreliable sources can help: (i) political scientists and journalists to analyse which topics of discussion are related to disinformation on a large scale (Bode & Vraga, 2015); (ii) social media platforms such as Twitter or Facebook to prevent the diffusion of potentially unreliable stories in the network (Castillo, Mendoza & Poblete, 2011; Conroy, Rubin & Chen, 2015; Shu et al., 2017); and (iii) psychologists to complement studies on personality analysis (Pennycook & Rand, 2018). The main contributions of our paper are as follows:

• We frame a novel binary classification task for early detection of users sharing content from unreliable news sources using diverse language features extracted from the aggregate of users’ original tweets;
• We evaluate a battery of traditional feature-based and neural predictive models that achieve up to 79.7 F1 score;
• We perform a qualitative analysis of our results to shed light into language use differences of users that diffuse content from unreliable or reliable news sources;

## Background and Related Work

### Disinformation in social media

Social media has become a primary platform for live-reporting (Engesser & Humprecht, 2015) with the majority of mainstream news media operating official accounts (e.g., @BBC and @Reuters on Twitter). However, social media platforms are also regarded as a fertile breeding ground for the diffusion of unverified, fabricated and misleading information due to its openness and popularity (Zubiaga et al., 2018a). This type of information is often referred to as misinformation.

Misinformation has been defined as an umbrella term to include any incorrect information that is diffused in social networks (Wu et al., 2019). On the other hand, disinformation is defined as the dissemination of fabricated and factually incorrect information with main aim to *deliberately deceive* its audience (Glenski, Weninger & Volkova, 2018b).

### Categorization of unreliable news sources

Unreliable news sources are categorized by their intention and the degree of authenticity of their content (Rubin, Chen & Conroy, 2015; Rashkin et al., 2017). Rubin, Chen & Conroy (2015) define three categories of deceptive news: (1) serious fabrications including unverified claims coupled with exaggerations and sensationalism; (2) large-scale hoaxes that are masqueraded as credible news which could be picked up and mistakenly disseminated; and (3) humorous fakes that present fabricated purposes with no intention to deceive. Rashkin et al. (2017) extended these three groups of misinformation into a more fine-grained classification:

• Propaganda news uses misleading information and writing techniques (Martino et al., 2019) to promote a particular agenda (Glenski, Weninger & Volkova, 2018a). Propaganda news sources that mostly share unreliable stories often aim to manipulate people’s opinions and influence election results posing a threat to political stability worldwide (Allcott & Gentzkow, 2017; Humprecht, 2018).
• Clickbait is defined as using exaggerated headlines for grabbing user attention and misleading public opinion (Glenski, Weninger & Volkova, 2018a).
• Conspiracy theories can be understood as a kind of distorted interpretation of real events from people with ulterior motives such as political and religious groups (Goertzel, 1994; Byford, 2011).
• Satire news commonly mimics professional news press, incorporating irony and illogical contents for humour purposes (Tandoc Jr., Lim & Ling, 2018; Burfoot & Baldwin, 2009).

Recent efforts on detecting and index unreliable news sources rely on crowdsourcing and experts ^1^ 1For example: http://www.fakenewswatch.com/, http://www.propornot.com, https://mediabiasfactcheck.com, etc.to annotate the reliability of the news media (Volkova et al., 2017; Baly et al., 2018; Glenski, Weninger & Volkova, 2018a).

### Previous work on combating online disinformation

Previous work on combating diffusion of disinformation in social media (Castillo, Mendoza & Poblete, 2011; Conroy, Rubin & Chen, 2015; Shu et al., 2017) has focused on characterizing the trustworthiness of (1) news sources (Dong et al., 2015; Baly et al., 2018); (2) news articles (Rashkin et al., 2017; Horne et al., 2018; Potthast et al., 2018; Pérez-Rosas et al., 2018); and (3) individual claims including news article headlines and rumors (Popat et al., 2016; Derczynski et al., 2017; Volkova et al., 2017; Zubiaga et al., 2018b; Thorne & Vlachos, 2018). Zhou et al. (2019) present a novel task for detecting the check-point which can early-detect a rumor propagated in a social network. Martino et al. (2019) develop models for detecting up to 18 writing techniques (e.g., loaded language, slogans, flag-waving, exaggeration, etc.) used in propaganda news. Similarly, Pathak & Srihari (2019) introduced a corpus of news articles related to US politics containing false assertions which are written in a compelling way. At the user level, social scientists and psychologists have utilised traditional methods, such as recruiting participants for online surveys and interviews, to explore cognitive factors which may influence people’s ability to distinguish fake news (Pennycook, Cannon & Rand, 2018). For instance, the lack of analytic thinking plays a vital role in recognition of misinformation (Pennycook & Rand, 2018). Previous data-driven studies include (1) analysing bots participation in social media discussion (Howard & Kollanyi, 2016) and distinguishing between automated and human accounts (Mihaylov & Nakov, 2016); (2) identifying user reactions (e.g., agreement, answer, appreciation, humor, etc.) to reliable/unreliable news posts (Glenski, Weninger & Volkova, 2018a); and (3) analyzing the demographic characteristics of users propagating unreliable news sources (Glenski, Weninger & Volkova, 2018b), e.g., low-income and low-educated people are more likely to propagate unreliable news sources on social networks.

In our paper, we tackle the problem of early detecting users who are likely to share post from unreliable news sources which is rather different to the focus of previous work on disinformation detection and analysis.

## Task Description

Our aim is the early detection of social media users that are likely to repost content from unreliable news sources before they actually share any other news items at all. To that end, we define a novel binary classification task for predicting whether a social media user will propagate news items from unreliable or reliable news sources using solely **language information**^2^. 2Note that one could use a user’s social network information but this is out of the paper’s scope because we are interested in analysing differences in language use between the two groups of users.

We assume a training set of *n* users *U* = {(*x*_1_, *y*_1_), …, (*x*_*n*_, *y*_*n*_)} where *x*_*i*_ is a vector representation of language information extracted from user’s *i* timeline consisting of posts up to the first repost of any news item, and *y*_*i*_ ∈ {reliable,unreliable } is an associated user label. Given *U*, we learn a function *f* that maps a new user *j* into one of the two categories $\hat {y}=f({x}_{j})$ using any suitable supervised machine learning algorithm.

We consider the posts up to the first share of any news item, ensuring that we only use prior information that is not linked to any news source. One could also introduce a cut-off in time or keep the top *k* posts but we choose to use all the available information possible. We opted to define a binary task (i.e., reliable vs. unreliable) rather than a fine-grained classification task (i.e., propaganda, hoax, clickbait, and reliable) because propagating any type of disinformation might be equally harmful. For similar reasons, we are not focusing on modeling the proportion of posts from reliable/unreliable sources in users’ Twitter Timeline.

## Data

At present, there is no existing dataset to model our predictive task. For the purposes of our experiments, we develop a new dataset of Twitter users who have retweeted posts from unreliable or reliable news sources. We opted for Twitter because the majority of accounts and posts are publicly available and it has been extensively used in related work (Volkova et al., 2017; Rashkin et al., 2017; Glenski, Weninger & Volkova, 2018a).

**Figure 1:**
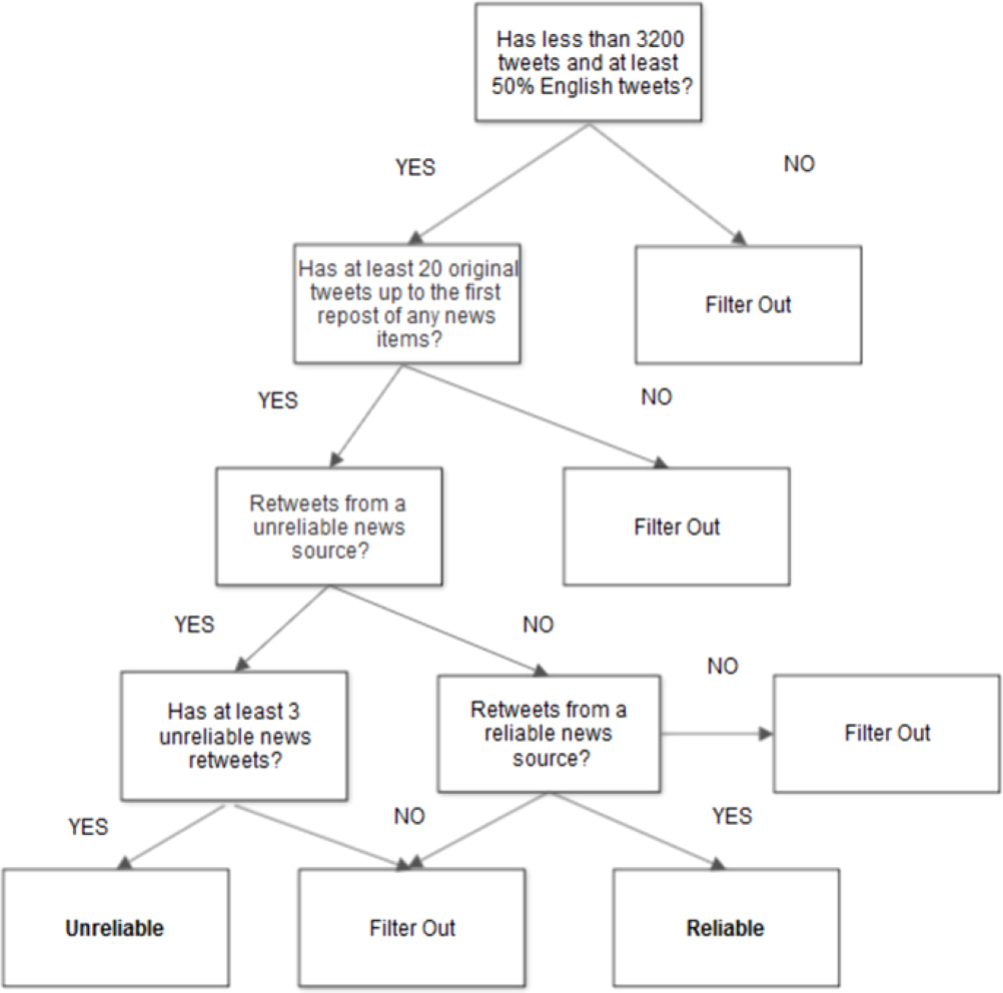
User filtering and labeling flowchart.

Our data collection process consists of three main steps (summarized in Fig. 1): (1) collection of posts from reliable and unreliable news sources; (2) collection of candidate users that have shared at least one of the posts collected during the first step; (3) assignment of users to the reliable and unreliable categories.

### Collecting posts from unreliable and reliable news sources

To identify users that have shared content from a particular news source, we first need to collect posts from reliable and unreliable news sources. For that purpose, we use a widely-used and publicly available list of *English news media* Twitter accounts provided by Volkova et al. (2017) and Glenski, Weninger & Volkova (2018a), which contains 424 English news media sources categorized in *unreliable* (satire, propaganda, hoax, clickbait) and *reliable*, following Rubin, Chen & Conroy (2015). For each news source, we retrieve the timeline (most recent 3,200 tweets) using the Twitter public API. We then filter out any retweets to ensure that we can collect only original posts from each Twitter account.

In this list, unreliable news sources (e.g., Infowars, Disclose.tv) have been annotated by digital journalism organisations (e.g., PropOrNot, fakenewswatch.com, etc.), while the reliable news media accounts (e.g., BBC, Reuters) have all been verified on Twitter and used in Glenski, Weninger & Volkova (2018a). Since satire news sources (e.g., The Onion, Clickhole) have humorous purposes (no desire to deliberately deceive (Rashkin et al., 2017)), we exclude them as in Glenski, Weninger & Volkova (2018b) resulting into 251 trusted and 159 unreliable sources. Note that the list does not exhaustively cover all the available sources but it is a representative sample for the purpose of our experiments. We also use the characterization of an entire news source as reliable/unreliable following Rashkin et al. (2017); Volkova et al. (2017) and not individual posts.

### Collecting Candidate Users

We retrieve an initial set of approximately 15,000 candidate users by looking into the most recent user accounts who have retweeted at least an original tweet from each news source. Due to the public Twitter API limits, we do not have access to user likes of news items. Based on the user profile information, we filter out users with more than 3,200 tweets due to the Twitter public API limits, since we need access to the entire timeline to decide the category the user belongs to (see section Labeling Users). For the remaining users, we collect their entire timeline (up to 3,200 tweets) and we filter-out any user with majority of non-English tweets (i.e., tweets labelled as ‘en’ or ‘en-gb’ by Twitter). Then for each user, we count the number of retweets from reliable and unreliable news sources respectively. Subsequently, we remove all user’s retweets (including tweets containing RT) and we keep only the tweets up to the first retweet of a news source for each user. Moreover, we only keep users with more than 10 original tweets.

### Labeling users

Our classification task is defined as the early detection of users posting unreliable news sources before they actually do it for the first time. Therefore, candidate users are assigned into two categories (*Unreliable, Reliable*):

• **Unreliable.** Users that have *reposted unreliable sources at least three times* (to ensure that this is a consistent behaviour) including the case *when a user has shared both reliable and unreliable sources* (modeling the ratio of unreliable/reliable it is out of the scope of early detection) are assigned to the unreliable class.
• **Reliable**. Users that have retweeted *only* reliable news sources are assigned to the reliable category.

Given that Twitter users can also share shortened URLs from unreliable news websites (e.g., http://www.infowars.com), we collect and expand all shortened URLs (e.g., ’https://t.co/example’) extracted from the posts of users labeled as reliable. We then remove all users who have shared any URLs from unreliable news websites. Our data collection process yielded a set of 6,266 users (3,468 and 2,798 for reliable and unreliable respectively) with a total of 1,356,480 tweets (see Table 1).

**Table 1:** Dataset statistics.

	**Unreliable**	**Reliable**
**Users**		
Total	2,798	3,468
**Tweets**		
Min	10	10
Max	2,600	2,613
Mean	172	252
Median	71	116
Total	481,199	875,281
**Tokens**		
Min	17	10
Max	37,576	251,030
Mean	1,796	2,779
Median	657	1,150
Total	5,024,341	9,713,595

### Text preprocessing

We pre-process all tweets from all users by first lowercasing text and then tokenizing. Furthermore, we remove any stop words^3^ 3We use the NLTK English stopwords list.and replace all URLs and @-mentions with url and usr markers respectively. See Table 1 for token statistics per user.

### Ethics

Previous work on the study of who spreads misinformation in social networks has used data collected through survey questionnaires (i.e., self-report data) and trace data (i.e., user-generated content) (Talwar et al., 2019; Chen et al., 2015; Shu et al., 2019; Hopp, Ferrucci & Vargo, 2020). We employ similar standard practices on studying social media user behavior. Our work has received approval from the University of Sheffield Research Ethics Committee (Ref. No 025470) and complies with Twitter data policy for research (https://developer.twitter.com/en/developer-terms/agreement-and-policy). Note that we will not share the data for non-research purposes.

## Methods

### SVM

We use Support Vector Machines (SVM) with an Radial Basis Function (RBF) kernel (Joachims, 2002) for all of our feature-based models which can be considered as baselines. We extract three types of language features: (1) Bag-Of-Words (BOW); (2) topics; and (3) Linguistic Inquiry and Word Count (LIWC), following a similar approach to recent work in computational social science (Rashkin et al., 2017; Pérez-Rosas et al., 2018; Zhang et al., 2018; Holgate et al., 2018):

• We use **BOW** to represent each user as a TF-IDF weighted distribution over a 20,000 sized vocabulary with the most frequent unigrams, bigrams and trigrams. We only consider n-grams appearing in more than five and no more than 40% of the total users.
• We also represent each user over a distribution of 200 generic word clusters (**Topics**^4^ 4Early experimentation with topic models did not yield highly coherent topics.) computed on a Twitter corpus and provided by Preoţiuc-Pietro, Lampos & Aletras (2015) for unveiling the thematic subjects that the users discuss.
• We finally represent each user over a distribution of 93 psycho-linguistic categories represented by lists of words provided by the Linguistic Inquiry and Word Count (**LIWC**) 2015 dictionary (Pennebaker, Francis & Booth, 2001).

We then train SVMs using the three types of features: **SVM-BOW**, **SVM-Topics** and **SVM-LIWC** individually and in combination (**SVM-All**).

### Avg-EMB

As our first neural model, we use a simple feed forward network (Avg-EMB) which takes as input the concatenation of all the tokenized tweets of a user. Words from users’ tweets are first mapped into embeddings to compute an average embedding which represents the textual content posted by a user. Subsequently, the average embedding is passed to the output layer with a sigmoid activation function for binary classification.

### BiGRU-ATT

Furthermore, we train a bidirectional Gated Recurrent Unit (Cho et al., 2014) with self-attention (Xu et al., 2015) (BiGRU-ATT).^5^ 5We also tested a Hierarchical Attention Network (Yang et al., 2016) achieving similar performance to BiGRU-ATT.The input is first mapped into word embeddings which are then passed through a BiGRU layer. A user content embedding is computed as the sum of the resulting context-aware embeddings weighted by the self-attention scores. The user content embedding is then passed to the output sigmoid layer.

### ULMFiT

The Universal Language Model Fine-tuning (ULMFiT) (Howard & Ruder, 2018) is a transfer learning approach that uses a Average-Stochastic Gradient Descent Weight-Dropped Long Short-Term Memory (AWD-LSTM) (Merity, Keskar & Socher, 2017) encoder pre-trained on a large corpus using a language modelling objective. Following the standard adaptation process of ULMFiT, we first fine-tune the AWD-LSTM on language modelling using our dataset, and then we adapt the classifier into our binary task by replacing the output layer. We finally fine-tune ULMFiT using the gradual unfreezing method proposed in Howard & Ruder (2018).

### T-BERT and H-BERT

Deep Bidirectional Transformers (BERT) (Devlin et al., 2018) is a state-of-the-art masked language model based on Transformer networks (Vaswani et al., 2017) pre-trained on large corpora, i.e., Books Corpus and English Wikipedia. Given the maximum input sequence length of BERT is 512, we first use a truncated version of BERT (T-BERT), which only takes the first 512 word pieces of each user as input. For our specific binary classification task, we add a fully-connected layer with a sigmoid activation on top of the user contextualized embedding obtained by passing input through BERT.

In order to take into account all the available textual information, we also employ a hierarchical version of BERT (H-BERT) since the majority of users’ concatenated tweets is longer than 512 word pieces. Here, each input is split into several 512 length word chunks. The output for each chunk is averaged into a single vector before is passed through the same output layer as in T-Bert.

### T-XLNet and H-XLNet

XLNet is a generalized autoregressive language model (Yang et al., 2019) similar to BERT which has achieved state-of-the-art performance in multiple NLP tasks. XLNet uses a perturbed language model objective instead of masked language model used in BERT. Similar to BERT-based models, we employ both truncated and hierarchical versions of XLNet (i.e., T-XLNet and H-XLNet respectively) adapting them to our task using sigmoid output layers.

## Results

### Experimental setup

We split our data into train (70%), development (10%), and test (20%) sets. The development set is used for tuning the hyper-parameters of the models.

Following a similar hyper-parameter tuning method to recent work in computational social science (Vempala & Preoţiuc-Pietro, 2019; Maronikolakis et al., 2020), we tune the penalty parameter *C* ∈ {10, 1e2, 1e3, 1e4, 1e5} and *n-gram range* ∈ {(1,1), (1,2), (1,3), (1,4)} of the SVMs, setting *C* = 1*e*4 and *n-gram range* =(1, 2). For BiGRU-ATT, we tune the GRU *hidden unit size* ∈ {50, 75, 100} and *dropout rate* ∈ {0.2, 0.5} observing that 50 and 0.5 perform best respectively. For Ave-EMB and BiGRU-ATT, we use Glove embeddings (Pennington, Socher & Manning, 2014) pre-trained on Twitter (*d* = 200). For all neural models, we use binary cross-entropy loss and the Adam optimizer (Kingma & Ba, 2015) with default learning rate 0.001 (except of the fine-tuning of ULMFiT, BERT and XLNet models where we use the original learning rates). We use a batch size of 8 for BERT, XLNet models and 64 for the rest of the neural models respectively.

We repeat the training and testing of each model three times by setting different random seeds and finally report the averaged macro precision, recall and F1-score. All dataset splits and random seeds will be provided for reproducibility.

### Prediction results

Table 2 presents the results of the SVM with all the feature combinations (BOW, Topics, LIWC and All) and the neural models.

**Table 2:** Macro precision - **P**, recall - **R** and F1-score - **F1** (mean ± standard deviation over three runs) for predicting whether a Twitter user belongs to the reliable or unreliable class.

**Model**	**P**	**R**	**F1**
**Baselines**			
**SVM**	
BOW	75.8 ± 0.0	75.9 ± 0.0	75.8 ± 0.0
Topics	71.8 ± 0.0	71.1 ± 0.0	71.2 ± 0.0
LIWC	69.8 ± 0.0	69.6 ± 0.0	69.6 ± 0.0
All	75.9 ± 0.0	75.8 ± 0.0	75.9 ± 0.0
**Neural Models**			
Avg-EMB	76.3 ± 1.2	75.3 ± 1.1	75.5 ± 1.2
BiGRU-ATT	78.0 ± 0.7	77.8 ±0.3	77.8 ± 0.5
ULMFiT	77.9 ± 0.2	77.2 ± 0.6	77.4 ± 0.5
T-BERT	**79.7**±0.2	**79.8**±0.1	**79.7**±0.1
H-BERT	79.5 ± 0.4	78.7 ± 0.6	78.9 ± 0.5
T-XLNet	79.6 ± 0.3	79.8 ±0.2	79.7 ± 0.3
H-XLNet	79.3 ± 0.3	78.9 ± 0.4	79.0 ± 0.3

In general, neural models achieve higher performance compared to feature-based models (SVM). Specifically, the T-BERT model achieves the highest F1 score overall (79.7) surpassing all the feature-based models as well as other neural network-based methods. This demonstrates that neural models can automatically unveil (non-linear) relationships between a user’s generated textual content (i.e., language use) in the data and the prevalence of that user retweeting from reliable or unreliable news sources in the future. The simpler neural network model, Avg-EMB achieves a lower F1 score (75.5) compared to the other neural models, i.e., BiGRU-ATT, BERT, XLNet and ULMFiT. This happens because the latter have more complex architectures and can effectively capture the relations between inputs and labels while the former ignores word order. Furthermore, ULMFit, BERT and XLNet models have been pre-trained on large external corpora so they can leverage this extra information to generalize better. Finally, we do not notice considerable differences in performance between the truncated and hierarchical versions of the transformer-based models (BERT and XNLNet) suggesting that a small amount of user generated content is enough for accurately predicting the correct user class.

Best single performing feature-based model is SVM-ALL (75.9). Moreover, SVM with BOW, Topics and LIWC achieve lower performance (75.8, 71.2 and 69.6 respectively).

### Error analysis

We performed an error analysis on the predictions of our best model, T-BERT. We notice that users in the unreliable class who are classified as reliable are those who repost from both reliable and unreliable sources. These users have an average of 40 future retweets from reliable news sources which is higher than the average number (31 retweets) in the entire dataset. Therefore, it is likely that such users use similar topics of discussion with reliable users. On the other hand, there is a of total 454 unreliable users who have no retweets from reliable sources in our dataset, interestingly, only four of them are classified wrongly. We also observe that it is harder for our model to classify correctly reliable users when they have only posted a small number of original tweets (e.g., 10-60).

### Linguistic analysis

Finally, we perform a linguistic feature analysis to uncover the differences in language use between users in the two classes, i.e., reliable and unreliable. For that purpose, we apply univariate Pearson’s correlation test to identify which text features (i.e., BOW, Topics and LIWC) are high correlated with each class following Schwartz et al. (2013). Tables 3, 4 and 5 display the top-10 n-grams, LIWC categories and Topics (represented by the most central words as in Preoţiuc-Pietro et al. (2015)) respectively. All Pearson correlations (r) presented in tables are statistically significant (*p* < 0.001).

**Table 3:** N-grams associated with unreliable and reliable categories sorted by Pearson’s correlation (*r*) between their normalized frequency and the labels (*p* < .001).

**n-grams**			
**Unreliable**	**r**	**Reliable**	**r**
war	0.140	school	0.150
media	0.137	gonna	0.133
government	0.135	myself	0.133
truth	0.133	wanna	0.131
israel	0.123	feel	0.131
liberal	0.122	excited	0.131
msm	0.121	mom	0.127
liberals	0.113	mood	0.122
muslim	0.113	okay	0.121
islam	0.112	rn	0.121

**Table 4:** Topics associated with unreliable and reliable categories sorted by Pearson’s correlation (*r*) between the topic normalized frequency and the labels. All correlations are significant (*p* < .001, *t*-test, Simes corrected).

**Topics**		
**#**	**Unreliable**	**r**
175	religious, colonialism, christianity, judaism, persecution, fascism, marxism, nationalism, communism, apartheid	0.244
118	#libya, libyan, libya’s, loyalists, palestinians, iran’s, gaddafi’s, al-qaeda, libya, repression	0.21
138	republican, democratic, gop, congressional, judiciary, hearings, abolishing, oppose, legislation, governors	0.196
106	allegations, prosecution, indictment, alleged, convicted, allegation, alleges, accused, charges, extortion	0.184
18	harper, congressman, abbott, mccain, cain, turnbull, spokesman, corbett, president, chairman	0.183
179	gov’t, govt, government, government’s, govt’s, privatisation, bureaucrats, draconian, safeguards, bureaucracy	0.173
160	latvian, bulgarian, croatian, turkish, malaysian, estonia, hungarian, basque, cypriot, romanian	0.166
196	govern, compromises, ultimately, unwilling, distrust, thereby, establish, assert, willingness, inaction	0.165
78	self-serving, hypocritical, moronic, idiocy, bigoted, blatant, reactionary, dismissive, uninformed, pandering	0.149
176	armed, gunmen, killings, suspected, bombings, police, detained, authorities, policemen, arresting	0.148
**#**	**Reliable**	**r**
120	physics, sociology, maths, biology, math, chem, calculus, geog, worksheet, worksheet	0.143
101	4 h, #naptime, #sleepy, 4hrs, 6hrs, #exhausted, #tired, 3 h, 3hrs, #sotired	0.14
2	tomorrows, tmw, tomorrow, tomor, tomrw, #hopefully, 4day, #tgif, arvo, tmrw	0.135
53	giggling, giggled, hysterically, squealing, sobbing, moaned, gasped, screaming, awkwardly, angrily	0.125
1	tights, cardigan, slacks, sleeveless, sweater, plaid, skirt, v-neck, leggings, skinnies	0.119
65	#foodtweets, #foodtweet, yummm, yummmm, #nomnom, spaghetti, sandwich, #yum, yummmmmm, #yummy	0.119
9	horribly, dreadfully, slighty, terribly, hungover, hungover, majorly, majorly, horrid	0.118
27	1:30, 6:15, 3:30, 8:45, 7:45, 4:30, 8:15, 9:45, 5:30, 2:30	0.116
166	chocolate, strawberry, choc, toffee, cinnamon, almond, parfait, butterscotch, choco, strawberries	0.112
33	b’day, birthday, birthdaaaay, birthdayyyyy, b-day, birthday, birthdayyyy, birthdaaay, bday, birfday	0.102

**Table 5:** LIWC features associated with unreliable and reliable categories sorted by Pearson’s correlation (*r*) between the normalized frequency and the labels (*p* < .001).

**LIWC**			
**Unreliable**	**r**	**Reliable**	**r**
*Analytic*	0.242	*Informal*	0.200
*Power*	0.203	*NetSpeak*	0.192
*Words* > 6*letters*	0.184	Word Count	0.129
*Space*	0.153	*Authentic*	0.093
*Drives*	0.140	*Ingest*	0.087
*Risk*	0.125	*Bio*	0.080
*Religion*	0.125	*Feel*	0.073
*Money*	0.117	*WordsPerSent*.	0.071
*Death*	0.105	*Leisure*	0.067
*Neg*.*Emotion*	0.097	*Time*	0.064

#### BOW

Table 3 shows the ten most correlated **BOW** features with each class. We observe that users reposting unreliable news sources in the future are more prevalent in tweeting about politics (note that we exclude user retweets in our study). For example, they use words related to the established political elite (e.g., *liberal, government, media, MSM*^6^) 6MSM is an Internet acronym for “mainstream media”.and Middle East politics (e.g., *islam, israel*). This may be partially explained by studies which find that people who are more ideologically polarized might be more receptive to disinformation (Marwick, 2018) and engage more with politics on social media (Preoţiuc-Pietro et al., 2017). Users using language similar to the language used by unreliable and hyperpartisan sources can be explained by the fact that these users might already consume news from unreliable sources but they have not reposted any of them yet (Potthast et al., 2018; Pennycook, Cannon & Rand, 2018).

Users belonging in the reliable news sources category use words related to self-disclosure and extraversion such as personal feelings and emotions (e.g., *mood, wanna, gonna, i’ll, excited*). Moreover, words such as *birthday, okay* denote more frequent interaction with other users, perhaps friends.

#### Topics

Table 4 shows the ten most correlated **topics** with each class. Topics related to politics such as political ideology (#138, #175), government (#179) and justice (#106) are correlated with users that will propagate unreliable sources, aligned with the n-grams analysis. We also observe a high correlation of such users with the topic related to impolite personal characterizations (#78). This corroborate results of a recent study that showed political incivility on Twitter is correlated to political polarization (Vargo & Hopp, 2017).

Users who will repost reliable sources discuss topics related to their day-to-day life such as education (#120), food (#65 and #166) and fashion (#1). Some topic words (e.g., sleep, exhausted and tired from #101) reveal that users emotional or physical states caused from work or study. In other words, these users tend to share more frequently information about their daily life, time and schedule (#101, #2 and #27).

#### LIWC

Table 5 shows the ten most correlated **LIWC** categories with each class. LIWC categories such as *Power* and *Drives* are more prevalent in users that will share unreliable sources. We also observe the difference in using casual language, e.g., *Netspeak* and *Informal* categories which are more often used by users that will share trusted sources.

## Conclusions

We have presented a new study on the early detection of users reposting unreliable news sources. We have created a new dataset with users labeled into the two categories, i.e., reliable and unreliable. For this binary classification task, a Transformer-based pretrained model (i.e., BERT) achieves up to 79.7 macro F1. Finally, our linguistic feature analysis unveiled the main characteristics and differences between language features (i.e., BOW, Topics and LIWC) in the two groups of users. In the future, we plan to extend this work by performing a fine-grained classification into hoax, propaganda and clickbait (Glenski, Weninger & Volkova, 2018b); and explore whether language and social network information are complementary.

##  Supplemental Information

10.7717/peerj-cs.325/supp-1 Supplemental Information 1 User and label

10.7717/peerj-cs.325/supp-2 Supplemental Information 2 SVM

10.7717/peerj-cs.325/supp-3 Supplemental Information 3 xlnet

10.7717/peerj-cs.325/supp-4 Supplemental Information 4 BERT

10.7717/peerj-cs.325/supp-5 Supplemental Information 5 ULMFit

10.7717/peerj-cs.325/supp-6 Supplemental Information 6 bigru attention

## Additional Information and Declarations

### Data Availability

The following information was supplied regarding data availability: The dataset are available in the Supplementary Files.
